# Supplementary material for: A polyomavirus peptide binds to the capsid VP1 pore and has potent antiviral activity against BK and JC polyomaviruses
Source: eLife. 2020 Jan 21;9:e50722. doi: 10.7554/eLife.50722 (PMC6974358; doi:10.7554/eLife.50722)
Supplement: Supplementary file 5. [file elife-50722-supp5.docx]

| **Key Resources Table** | | | | |
| --- | --- | --- | --- | --- |
| **Reagent type (species) or resource** | **Designation** | **Source or reference** | **Identifiers** | **Additional information** |
| Primary cells (*Homo sapiens*) | RPTE | ATCC | PCS-400-010 | Primary renal proximal tubule epithelial cells; sex and age batch-specific |
| cell line (*Homo sapiens*) | HEK-293 | ATCC | CRL-1573; RRID:CVCL_0045 |  |
| cell line (*Cercopithecus aethiops*) | COS-7 | ATCC | CRL-1651; RRID:CVCL_0224 |  |
| cell line (*Spodoptera frugiperda*) | Sf9 | Expression Systems | 94-001F; RRID:CVCL_0549 |  |
| strain, strain background (*Escherichia coli*) | XL10-Gold | Agilent | Cat # 200315 |  |
| strain, strain background (*Escherichia coli*) | BL21 Star (DE3) | Invitrogen | Cat # C601003 |  |
| strain, strain background (*Escherichia coli*) | 10-beta | NEB | Cat # C3019I |  |
| recombinant DNA reagent) | pBKV (35-1) | ATCC | 45026 | pBR322 backbone containing BKV ST1 genome (Genbank: J02039) |
| recombinant DNA reagent) | pM1TC | Walter Atwood | Genbank: J02227 | JCV genotype Ia isolate Mad1 cloned into pBR322 backbone |
| antibody | P8D11 (Human monoclonal) | This paper |  | Anti-BKV neutralizing antibody |
| antibody | Anti-BKV VP1 (Mouse monoclonal) | This paper |  | IF(1:500) |
| antibody | anti-SV40 T-antigen (Mouse monoclonal) | EMD Millipore | PAb416; RRID:AB_10682473 | IF(1:200) |
| antibody | anti-SV40 VP1 (Rabbit polyclonal) | Abcam | Cat # ab53977; RRID:AB_946338 | IF(1:500), IB (1:1000) |
| antibody | anti-SV40 VP2/3 (Rabbit polyclonal) | Abcam | Cat# ab53983; RRID:AB_946339 | IF(1:1000), IB (1:1000) |
| antibody | anti-GRP78 BiP (Rabbit polyclonal) | Abcam | Cat#: ab21685; RRID:AB_2119834 | IB (1:1000) |
| antibody | anti-Hsp90 antibody [D7a] (Mouse monoclonal) | Abcam | Cat#: ab59459; RRID:AB_942030 | IB (1:1000) |
| antibody | Anti-biotin (Rabbit polyclonal) | Abcam | Cat#: ab53494; RRID:AB_867860 | IF(1:750) |
| antibody | Goat anti-Mouse IgG (H+L) Cross-Adsorbed Secondary Antibody, Alexa Fluor 594 | Invitrogen | A-11005; RRID:AB_2534073 | IF(1:1000) |
| antibody | Goat anti-Rabbit IgG (H+L) Highly Cross-Adsorbed Secondary Antibody, Alexa Fluor 488 | Invitrogen | A-11034; RRID:AB_2576217 | IF(1:1000) |
| antibody | Goat anti-Mouse IgG (H+L) Cross-Adsorbed Secondary Antibody, Alexa Fluor 647 | Invitrogen | A-21235; RRID:AB_2535804 | IF(1:1000) |
| antibody | Goat anti-Rabbit IgG (H+L) Highly Cross-Adsorbed Secondary Antibody, Alexa Fluor 594 | Invitrogen | A-11037; RRID:AB_2534095 | IF(1:1000) |
| antibody | IRDye 800CW Goat anti-Rabbit IgG Secondary Antibody | Li-COR | P/N: 925-32211; RRID:AB_2651127 | IB (1:12,000) |
| antibody | IRDye 680RD Goat anti-Mouse IgG Secondary Antibody | Li-COR | P/N: 925-68070; RRID:AB_2651128 | IB (1:12,000) |
| peptide, recombinant protein | D1_min_ | This paper | Peptide | Ac-APQWMLPLLLGLY-NH_2_ |
| peptide, recombinant protein | D1_22_ | This paper | Peptide | APGGANQRTAPQWMLPLLLGLY |
| peptide, recombinant protein | biotin-D1_22_ | This paper | Peptide | biotin-GGGGAPGGANQRTAPQWMLPLLLGLY |
| peptide, recombinant protein | TAT-D1_min_ | This paper | Peptide | GRKKRRQRRR-PEG2-APQWMLPLLLGLY-NH2 |
| peptide, recombinant protein | D1_min_-TAT | This paper | Peptide | Ac-APQWMLPLLLGLY-PEG2-GRKKRRQRRR |
| sequenced-based reagent | VP1_P232S_F | This paper | PCR primers | caggaggggaaaatgtttccccagtacttcat |
| sequenced-based reagent | VP1_P232S_R | This paper | PCR primers | cacatgaagtactggggaaacattttcccctcctg |
| sequenced-based reagent | VP1_P232L_F | This paper | PCR primers | caggaggggaaaatgttctcccagtacttcat |
| sequenced-based reagent | VP1_P232L_R | This paper | PCR primers | cacatgaagtactgggagaacattttcccctcctg |
| sequenced-based reagent | VP1_P232I_F | This paper | PCR primers | caggaggggaaaatgttatcccagtacttcat |
| sequenced-based reagent | VP1_P232I_R | This paper | PCR primers | cacatgaagtactgggataacattttcccctcctg |
| sequenced-based reagent | VP1_V234S_F | This paper | PCR primers | gaaaatgttcccccatcacttcatgtgaccaac |
| sequenced-based reagent | VP1_V234S_R | This paper | PCR primers | gtgttggtcacatgaagtgatgggggaacatt |
| sequenced-based reagent | VP1_V234L_F | This paper | PCR primers | gaaaatgttcccccattacttcatgtgaccaac |
| sequenced-based reagent | VP1_V234L_R | This paper | PCR primers | gtgttggtcacatgaagtaatgggggaacatt |
| sequenced-based reagent | VP1_V234I_F | This paper | PCR primers | gaaaatgttcccccaatacttcatgtgaccaac |
| sequenced-based reagent | VP1_V234I_R | This paper | PCR primers | gtgttggtcacatgaagtattgggggaacatt |
| sequenced-based reagent | ΔVP2_F | This paper | PCR primers | gtatttccaggttcataggtgctgctctagcacttttgggggac |
| sequenced-based reagent | ΔVP2_R | This paper | PCR primers | gagcagcacctatgaacctggaaatacaaaaaaaaagggattac |
| sequenced-based reagent | ΔVP3_F | This paper | PCR primers | gcaatcaggcatagctttggaattgtttaacccagatgagtac |
| sequenced-based reagent | ΔVP3_R | This paper | PCR primers | ccaaagctatgcctgattgctgatagaggcctacagtggaaac |
| sequenced-based reagent | VP2_P291A_F | This paper | PCR primers | caaagaactgctgctcaatggatgttgcctttacttctaggcc |
| sequenced-based reagent | VP2_P291A_R | This paper | PCR primers | catccattgagcagcagttctttgattagcacctcctgg |
| sequenced-based reagent | VP2_W293A_F | This paper | PCR primers | ctgctcctcaagcgatgttgcctttacttctaggcctgtac |
| sequenced-based reagent | VP2_W293A_R | This paper | PCR primers | ggcaacatcgcttgaggagcagttctttgattagcacctcc |
| sequenced-based reagent | VP2_L297A_F | This paper | PCR primers | gatgttgcctgcacttctaggcctgtacgggactgtaacac |
| sequenced-based reagent | VP2_L297A_R | This paper | PCR primers | caggcctagaagtgcaggcaacatccattgaggagcagttc |
| sequenced-based reagent | VP2_Y302A_F | This paper | PCR primers | ctaggcctggccgggactgtaacacctgctcttgaagcatg |
| sequenced-based reagent | VP2_Y302A_R | This paper | PCR primers | gttacagtcccggccaggcctagaagtaaaggcaacatccattg |
| sequenced-based reagent | GAPDH PrimeTime primer set | IDT | Hs.PT.39a.22214836 |  |
| sequenced-based reagent | CXCL10 PrimeTime primer set | IDT | Hs.PT.58.3790956.g |  |
| sequenced-based reagent | IFNA2 PrimeTime primer set | IDT | Hs.PT.58.24294810.g |  |
| sequenced-based reagent | IFNB1 PrimeTime primer set | IDT | Hs.PT.58.39481063.g |  |
| sequenced-based reagent | MX1 PrimeTime primer set | IDT | Hs.PT.58.40261042 |  |
| sequenced-based reagent | OAS1 PrimeTime primer set | IDT | Hs.PT.58.2338899 |  |
| sequenced-based reagent | STAT1 PrimeTime primer set | IDT | Hs.PT.58.15049687 |  |
| commercial assay or kit | Recombinant Human IFN-β | Peprotech | Cat # 300-02BC |  |
| commercial assay or kit | RNeasy Mini Kit | Qiagen | Cat # 74104 |  |
| commercial assay or kit | QIAquick PCR Purification Kit | Qiagen | Cat # 28106 |  |
| commercial assay or kit | SuperScript IV VILO Master Mix | Invitrogen | Cat # 11756050 |  |
| commercial assay or kit | Fast SYBR Green Master Mix | Applied Biosystems | Cat # 4385610 |  |
| commercial assay or kit | CellTiter-Glo Luminescent Cell Viability Assay | Promega | Cat # G7570 |  |
| commercial assay or kit | Dynabeads M-280 Streptavidin | Invitrogen | Cat # 11205D |  |
| commercial assay or kit | InstantBlue Protein Stain | Expedeon | Cat # ISB1L |  |
| commercial assay or kit | Lipofectamine 2000 Transfection Reagent | Invitrogen | Cat # 11668027 |  |
| commercial assay or kit | Lipofectamine 3000 Transfection Reagent | Invitrogen | Cat # L3000008 |  |
| commercial assay or kit | Bac-to-Bac Vector Kit | Gibco | Cat # 10360014 |  |
| commercial assay or kit | AlphaScreen Protein A Acceptor beads | PerkenElmer | Cat # 6760137M |  |
| commercial assay or kit | Sensor Chip SA | GE Healthcare | Cat # BR-1005-31 |  |
| software, algorithm | CellProfiler | *Kamentsky et al.* 2011 | v2.1.2; RRID:SCR_007358 |  |
| software, algorithm | R | R Core Team | v3.5.1; RRID:SCR_001905 |  |
| software, algorithm | XLFit | IDBS | v5.5.0.5 |  |
| software, algorithm | Fiji | Schindelin *et al*. 2012 | Built on ImageJ v1.52b; RRID:SCR_002285 |  |
| software, algorithm | Biacore Evaluation Software | GE Healthcare | v2.0; RRID:SCR_015936 |  |
| other | DAPI stain | Calbiochem | Cat # 508741 | 1.67 µg/mL |
